# Controlled Formation of Skyrmion Bags

**DOI:** 10.1002/adma.202501250

**Published:** 2025-04-21

**Authors:** Lisa‐Marie Kern, Vladyslav M. Kuchkin, Victor Deinhart, Christopher Klose, Themistoklis Sidiropoulos, Maike Auer, Simon Gaebel, Kathinka Gerlinger, Riccardo Battistelli, Steffen Wittrock, Tamer Karaman, Michael Schneider, Christian M. Günther, Dieter Engel, Ingo Will, Sebastian Wintz, Markus Weigand, Felix Büttner, Katja Höflich, Stefan Eisebitt, Bastian Pfau

**Affiliations:** ^1^ Max Born Institute for Nonlinear Optics and Short Pulse Spectroscopy 12489 Berlin Germany; ^2^ Department of Physics and Materials Science University of Luxembourg Luxembourg L‐1511 Luxembourg; ^3^ Ferdinand‐Braun‐Institut (FBH) 12489 Berlin Germany; ^4^ Helmholtz‐Zentrum Berlin für Materialien und Energie GmbH 14109 Berlin Germany; ^5^ Experimental Physics V Center for Electronic Correlations and Magnetism University of Augsburg 86159 Augsburg Germany; ^6^ Technische Universität Berlin Zentraleinrichtung Elektronenmikroskopie 10623 Berlin Germany; ^7^ Technische Universität Berlin Institut für Optik und Atomare Physik 10623 Berlin Germany

**Keywords:** higher‐order skyrmions, ion irradiation, laser‐induced nucleation, skyrmion bags, X‐ray imaging

## Abstract

Topologically non‐trivial magnetic solitons are complex spin textures with a distinct single‐particle nature. Although magnetic skyrmions, especially those with unity topological charge, have attracted substantial interest due to their potential applications, more complex topological textures remain largely theoretical. In this work, the stabilization of isolated higher‐order skyrmion bags beyond the prototypical π‐skyrmion in ferromagnetic thin films is experimentally demonstrate, which has posed considerable challenges to date. Specifically, controlled generation of skyrmionium (2π‐skyrmion), target skyrmion (3π‐skyrmion), and skyrmion bags (with variable topological charge) are achieved through the introduction of artificially engineered anisotropy defects via local ion irradiation. They act as preferential sites for the field‐ or laser‐induced nucleation of skyrmion bags. Remarkably, ultrafast laser pulses achieve a substantially higher conversion rate transforming skyrmions into higher‐order skyrmion bags compared to their formation driven by magnetic fields. High‐resolution x‐ray imaging enables direct observation of the resulting skyrmion bags. Complementary micromagnetic simulations reveal the pivotal role of defect geometry–particularly diameter–in stabilizing closed‐loop domain textures. The findings not only broaden the experimental horizon for skyrmion research, but also suggest strategies for exploiting complex topological spin textures within a unified material platform for practical applications.

## Graphical abstract

This work demonstrates stabilization of complex magnetic skyrmion bags in ferromagnetic thin films through precisely engineered anisotropy defects using ion irradiation. The researchers achieve controlled field‐ and laser‐induced generation of skyrmionia, target skyrmions, and higher‐order skyrmion bags at room temperature. This reliable platform opens opportunities for studying the dynamics of these topological textures and their implementation in advanced spintronic devices.

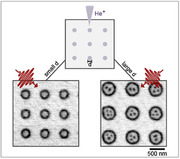

## 1 Introduction

Topologically non‐trivial magnetic textures are distinguished by their unique single‐particle character, which has attracted considerable interest in both fundamental and applied spintronics research.^[^ ^1^, ^2^ ^]^ These magnetic solitons remain localized within specific regions and cannot transform into textures with a different topology without violating the continuity of the magnetization field.^[^ ^3^ ^]^ Among these textures, the magnetic skyrmion with topological charge *Q* = ±1 (also known as π‐skyrmion) serves as the prototypical, most studied example.^[^ ^2^ ^]^ The π‐skyrmion has a planar geometry and is characterized by a smooth 180 degree rotation of the magnetization vector from out‐of‐plane spin‐up at the core of the skyrmion to the spin‐down direction at its outside, or vice versa.^[^ ^4^, ^5^ ^]^ This spin configuration has been observed in a plethora of host materials, including B20 helimagnets,^[^ ^6^, ^7^ ^]^ multiferroic material,^[^ ^8^ ^]^ ferromagnetic multilayers,^[^ ^9^, ^10^, ^11^, ^12^, ^13^, ^14^ ^]^ ferrimagnets,^[^ ^15^ ^]^ and van‐der‐Waals (vdW) heterostructures.^[^ ^16^, ^17^, ^18^ ^]^

A key advantage of isolated π‐skyrmions is their mobility in thin films, even at room temperature. Skyrmions can be efficiently displaced by spin‐orbit torque (SOT).^[^ ^10^, ^13^, ^14^, ^19^ ^]^ Their creation has been demonstrated also by SOT^[^ ^13^, ^15^, ^20^ ^]^ and ultrashort laser pulses.^[^ ^21^, ^22^, ^23^, ^24^ ^]^ Recent advancements in ion‐based materials engineering have further improved spatial control over individual skyrmion positions and trajectories.^[^ ^25^, ^26^, ^27^, ^28^ ^]^ Altogether, this advanced level of understanding and control of the π‐skyrmion led to the development of various device concepts,^[^ ^29^ ^]^ including examples in reservoir computing,^[^ ^30^, ^31^, ^32^, ^33^ ^]^ and racetrack memory devices.^[^ ^27^, ^34^, ^35^ ^]^

Beyond the simple winding of spins in π‐skyrmions, a wide variety of more complex topological spin textures has been identified.^[^ ^36^, ^37^ ^]^ These textures can be classified based on their dimensional complexity into three groups.

First, additional complexity arises from more intricate radial spin windings. This class of topological spin textures is known as skyrmion bags with arbitrary topological charge. They include the axis symmetric configurations of *k*π‐skyrmions,^[^ ^36^ ^]^ e.g., the skyrmionium (2π‐skyrmion) and the target skyrmion (3π‐skyrmion). Additionally, skyrmion bags encompass non‐axis symmetric configurations with arbitrary topological charge *Q* > 1^[^ ^18^, ^38^, ^39^, ^40^ ^]^ as well as tailed skyrmions.^[^ ^41^, ^42^ ^]^ Second, spin configurations may become more complex through azimuthal variations, manifesting as spin winding patterns within domain walls.^[^ ^43^ ^]^ Finally, even greater complexity emerges when spin textures extend into the third dimension, as observed in skyrmion tubes,^[^ ^44^, ^45^ ^]^ chiral bobbers,^[^ ^46^ ^]^ cocoons,^[^ ^47^, ^48^ ^]^ or hopfions.^[^ ^49^ ^]^

Despite their theoretical promise, experimental studies of these complex topological textures have been severely limited by challenges in their controlled creation and manipulation. While π‐skyrmions can be readily synthesized in various material systems, the controlled generation of higher‐order topological textures has proven exceptionally challenging. As a result, our understanding of their dynamics and potential applications has remained largely confined to theoretical predictions. This limitation has created a significant gap between the theoretical possibilities of complex topological textures and their practical implementation in functional devices.

In this work, we demonstrate a reliable method for creating and controlling skyrmion bags in an open, laterally unconfined thin‐film geometry. Our method represents a significant advance over previous approaches, which were limited to highly confined and topographically structured geometries^[^ ^11^, ^50^, ^51^, ^52^ ^]^ or relied on rare, stochastic events for texture formation.^[^ ^40^, ^53^, ^54^ ^]^ We demonstrate the creation of skyrmion bags in a well‐established ferromagnetic multilayer system, previously optimized for conventional skyrmion devices, enabling direct integration with existing spintronic technologies. The material system allows for both spin‐orbit torque and optical manipulation of the textures. Our approach is based on introducing nanometer‐scale anisotropy‐engineered defects, precisely positioned through localized helium‐ion irradiation.^[^ ^27^ ^]^ We achieve precise control over their topological charge and synthesize the skyrmionium (also known as 2π‐skyrmion), the target skyrmion (also known as 3π‐skyrmion), and skyrmion bags with higher topological charge up to *Q* = 4. This unprecedented level of control opens new possibilities for studying the dynamics of complex topological textures and provides a robust platform for their potential implementation in practical applications.

## 2 Skyrmion Bags

Complex topological spin textures can be classified by their topological charge Q (also known as the *skyrmion number*):^[^ ^1^ ^]^

(1) $$Q=\frac{1}{4\pi }\int_{S}m\left(r\right)\cdot \left[\partial_{x}m\left(r\right)\times \partial_{y}m\left(r\right)\right]dS$$

with the magnetization vector **m** = (sin θcos ϕ, sin θsin ϕ, cos θ) and the position vector **r** = (ρcos Φ, ρsin Φ, *z*). Here, θ = θ(ρ) denotes the polar angle as a function of the radius ρ, and ϕ(Φ) = ξΦ + δ is the azimuthal angle, shaping the in‐plane component of the magnetization. The topological charge *Q* can then be expressed as:^[^ ^1^, ^55^ ^]^

(2) $$Q=-\frac{1}{4\pi }{\left[\cos\theta \right]}_{\rho =0}^{\rho =\infty }\cdot {\left[\phi \right]}_{\Phi =0}^{\Phi =2\pi }=p\cdot \xi$$

with $p=-\frac{1}{2}{\left[\cos\theta \right]}_{\rho =0}^{\rho =\infty }$ denoting the polarity of the topological spin texture and $\xi =\frac{1}{2\pi }{\left[\phi \right]}_{\Phi =0}^{\Phi =2\pi }$ describing the vorticity or winding number of the magnetization. The polarity *p* and the vorticity ξ together define the topological charge of the skyrmionic texture, whereas the helicity δ does not contribute to the topological charge and solely affects the smooth canting of neighboring spins in the texture. The topological charge of these complex states can be straightforwardly computed relying on (1), or alternatively by extending definition (2) as explained in detail in Ref. [41].

The 2π‐skyrmion can be thought of as a soliton which is composed of two skyrmions with the same vorticity ξ but opposite polarity *p*, resulting in an outer skyrmion ring encircling an inner skyrmion with reversed polarity *p*.^[^ ^37^ ^]^ The texture can also be considered as an empty skyrmion bag (*S*(0)‐bag). In the context of applications, the 2π‐skyrmion is particularly promising as it is unaffected by the skyrmion Hall effect due to its compensated topological charge.^[^ ^39^, ^56^, ^57^, ^58^ ^]^ This compensation may allow it to move along a straight path, potentially reaching higher velocities and exhibiting greater robustness compared to the π‐skyrmion. Recent experimental evidence for a skyrmionium has been demonstrated in a magnetic topological insulator,^[^ ^59^ ^]^ in 2D van der Waals magnets,^[^ ^18^, ^40^, ^53^ ^]^ and after laser‐induced formation in a ferrimagnetic alloy.^[^ ^60^ ^]^ However, to explore application‐relevant dynamics and test theoretically suggested models, it is essential to stabilize the skyrmionium in magnetic materials that support spin‐torque driven motion, such as ferromagnetic thin films.^[^ ^61^, ^62^ ^]^

More complex textures are denoted as *S*(*Q*)‐bags.^[^ ^36^, ^38^, ^63^ ^]^ In these configurations, an outer closed‐loop domain encloses several inner skyrmions, with the number of inner skyrmions determining the topological charge of the texture. *S*(1)‐bags are commonly referred to as 3π‐skyrmions, which have already been observed in various materials, including ferromagnetic multilayers,^[^ ^54^ ^]^ though mostly within the confined space of topographically structured nanopillars.^[^ ^11^, ^50^, ^51^ ^]^ Higher‐order skyrmion bags, *S*(*Q* > 1), exhibit notable properties, including the high tunability of topological charge with inner skyrmions as new degrees of freedom,^[^ ^36^, ^52^ ^]^ the presence of eigenmodes^[^ ^64^ ^]^ and unique dynamics.^[^ ^39^, ^65^, ^66^ ^]^ From an applications perspective, these textures offer potential as high‐density information carriers, promising faster operation and reduced energy consumption in spintronic devices.^[^ ^37^, ^67^ ^]^ Recent experimental studies have observed certain skyrmion bags confined by other spin textures in FeGe monocrystals,^[^ ^68^ ^]^ and in 2D van der Waals magnets.^[^ ^53^ ^]^

However, the nucleation process of skyrmion bags has so far been stochastic. In particular, a precise positioning of skyrmion bags with nanometer spatial control and the emergence of a specific type of skyrmion bag could not be predefined. Thus, a reliable experimental protocol for generating isolated complex skyrmion states remained elusive.

A key challenge is achieving stable configurations, as these complex spin textures typically do not represent the system's ground state^[^ ^37^, ^53^ ^]^ or rarely appear as a stochastic representation within a highly degenerate domain pattern.^[^ ^40^, ^53^, ^54^ ^]^ Stabilization frequently requires spatial confinement^[^ ^11^, ^69^ ^]^ to induce strong edge effects,^[^ ^50^ ^]^ a condition that has limited experimental exploration and that is in stark contrast to the relatively straightforward formation of π‐skyrmions.^[^ ^2^ ^]^

## 3 Results

**Figure 1:**
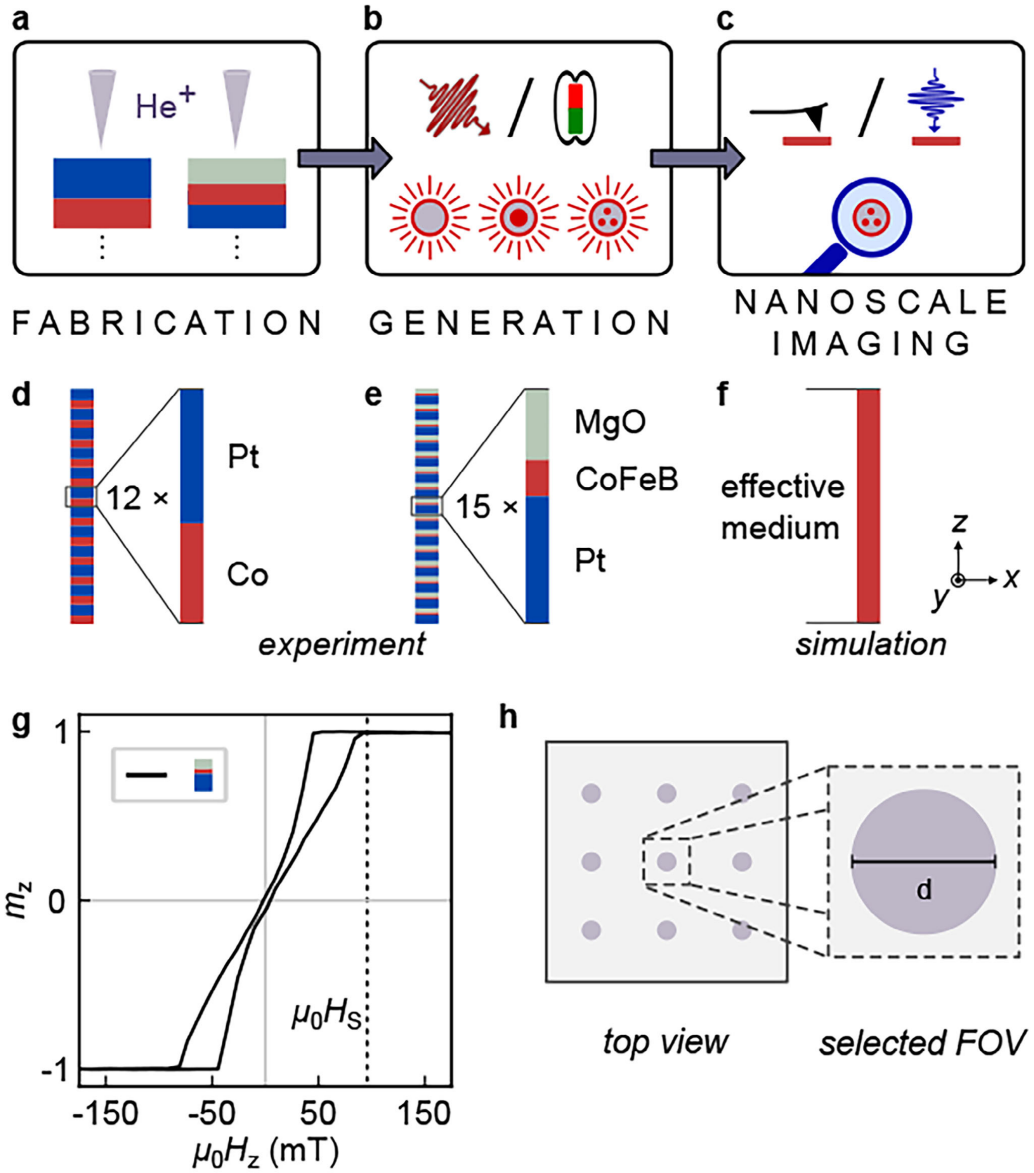
Experimental design: Creation, control, and observation of skyrmion bags. a) Nanofabrication including anisotropy‐engineering, b) generation of ultrafast laser‐ and magnetic‐field‐induced higher‐order skyrmions (left/right), and c) nanoscale observation via high‐resolution imaging using magnetic force microscopy and scanning transmission x‐ray microscopy (left/right). d–f) side view: Multilayer sample design for d) [Co/Pt] and e) [Pt/CoFeB/MgO] in experiment and f) the single simulated layer. g) Magnetic hysteresis of [Pt/CoFeB/MgO] measured via magneto‐optical Kerr effect (MOKE) microscopy. h) *top view*: Anisotropy‐engineered array of disks (purple) fabricated via focused helium ion irradiation.

Our experiment consists of three steps (**Figure** **1**a–c): I) fabricating ferromagnetic thin films with tailored perpendicular magnetic anisotropy (PMA) defects by helium ion irradiation, II) nucleating higher‐order skyrmionic textures through single laser pulses or magnetic‐field sweeps, and III) imaging these configurations at the nanoscale using magnetic force microscopy (MFM) or scanning transmission x‐ray microscopy (STXM). In each figure in this paper, we indicate the material, the skyrmion nucleation method, and the imaging method by pictograms introduced in Figure 1a–c.

**Figure 2:**
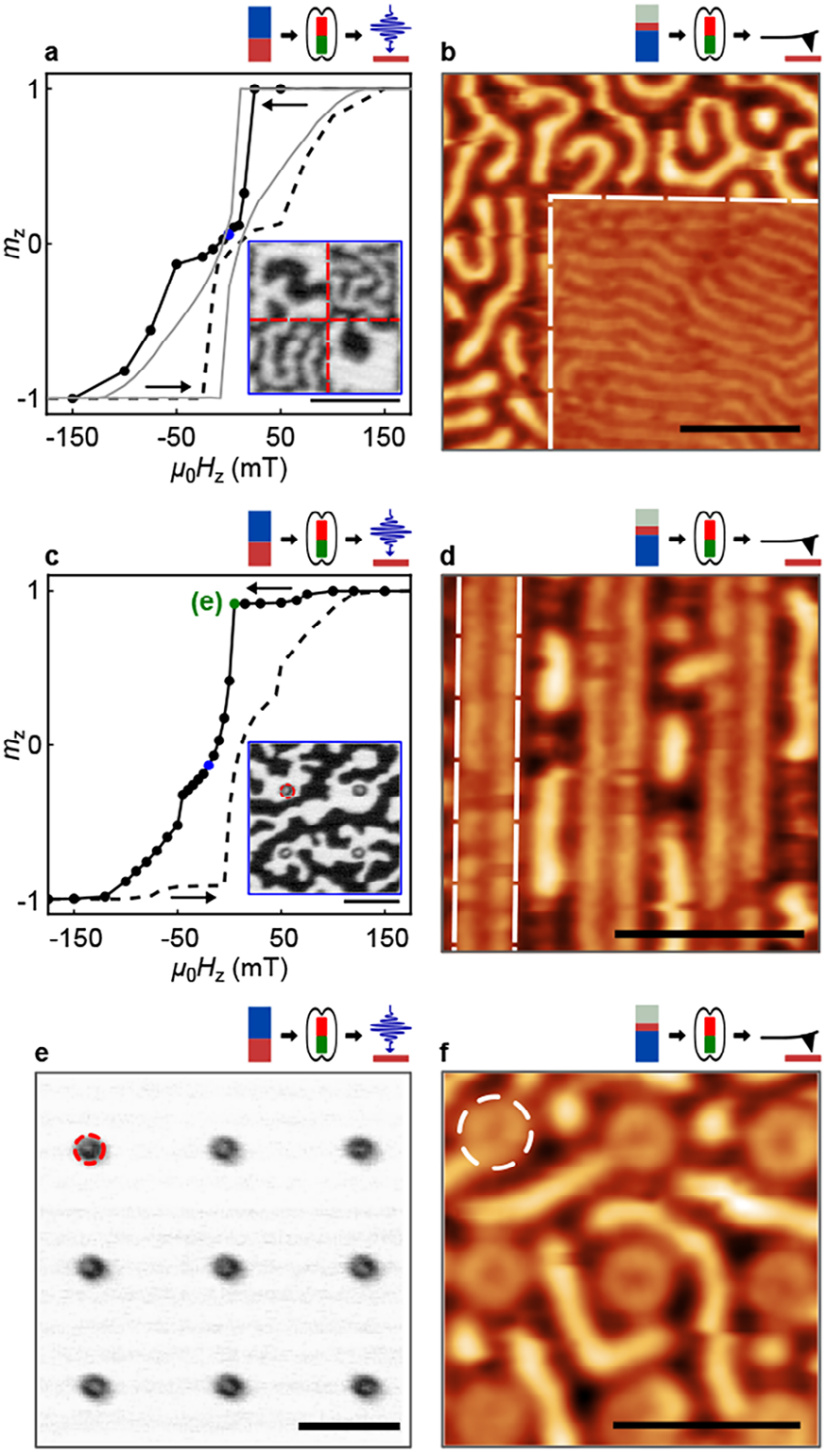
From domains to rings: Geometrical aspect of anisotropy‐engineering. a) Magnetic hysteresis of an ion‐irradiated checkerboard pattern (black curve) with 850 nm side length and ion dose 15 ions/nm^2^, calculated from magnetization images, and MOKE‐hysteresis of the pristine film (gray curve). The inset displays a representative STXM image at µ_0_ *H*_z_ = 0 mT. b) Magnetic force micrograph showing reduced domain width in irradiated square of 10μm side length and ion dose 250 ions/nm^2^ at µ_0_ *H*_z_ = 0 mT. c) Magnetic hysteresis of an ion‐irradiated array of disks with *d*_disk_ = 380 nm and ion dose 25 ions/nm^2^, calculated from magnetization images. The inset displays a representative STXM image at µ_0_ *H*_z_ = −15 mT. d) MFM image of self‐alignment of domains along predefined stripes of 300 nm width and ion dose 150 ions/nm^2^ at µ_0_ *H*_z_ = 0 mT. e,f) Closed‐loop domain formation inside disk shapes of STXM image in (e) (*d*_disk_ = 380 nm, 25 ions/nm^2^) at µ_0_ *H*_z_ = 5 mT and MFM image in (f) (*d*_disk_ = 350 nm, 250 ions/nm^2^) at µ_0_ *H*_z_ = 0 mT. All patterns except for (b) are irradiated in an area of 20μm × 20μm. Ion‐irradiated regions are exemplarily indicated by a dashed line. The left panels (a,c,e) show ion‐irradiated [Co/Pt], the right panels (b,d,f) display ion‐irradiated [Pt/CoFeB/MgO]. Pictograms highlight measured material, nucleation and imaging methods. Scalebars correspond to 1μm.

We prepare two types of ferromagnetic thin films: the symmetric Ta(3)/[Co(0.6)/Pt(0.8)]_× 12_/Ta(2), in the following referred to as [Co/Pt] (Figure 1d), and the asymmetric Ta(3)/Pt(4)/[Pt(2.5)/Co_60_Fe_24_B_16_(0.72)/MgO(1.4)]_× 15_/Pt(2), in the following referred to as [Pt/CoFeB/MgO] (Figure 1e). The magnetic hystereses for the non‐modified [Co/Pt] and [Pt/CoFeB/MgO] are presented in **Figure** **2**a (gray curve) and Figure 1g, respectively.

We support our experimental findings with micromagnetic simulations. In the simulations, the multilayers are modeled as a single layer with averaged material parameters according to the effective medium approach,^[^ ^10^ ^]^ as schematically depicted in Figure 1f (see Experimental Section for details on micromagnetic modeling).

As a key experimental step, we predefine nucleation sites by introducing nanometer sized anisotropy‐engineered regions via local helium‐ion irradiation, following previous work on focused ion‐irradiation.^[^ ^26^, ^27^, ^28^ ^]^ Anisotropy‐engineering of tailored defects is performed employing the ZEISS Orion NanoFab He^+^‐FIB system. We prepare circular areas with various diameters inside the layer, as schematically presented for an array of irradiated circular areas (purple) with diameter *d*_disk_ in Figure 1h, leaving the topography of the samples unaffected. For simplicity, we denote these circular‐shaped regions as disks. We identified a suitable ion dose range of 10–50 ions/nm^2^ for [Co/Pt] and a broader range of 25–400 ions/nm^2^ for [Pt/CoFeB/MgO], similar to our previous work.^[^ ^27^ ^]^ Within this range, the magnetic film retains its perpendicular magnetic anisotropy, albeit at a reduced level, significantly influencing the domain morphology, as outlined in the next section. Note that the required helium ion doses are significantly increased for the much thicker [Pt/CoFeB/MgO] compared to [Co/Pt]. The tailored defects are modeled in the simulation as regions of reduced anisotropy constant *K* (see Experimental Section for more details).

### 3.1 Tailoring Domain Morphology

First, we investigated how ion irradiation influences the formation of domain patterns in our materials in general. In the [Co/Pt] multilayer, we created a checkerboard pattern (Figure 2a), and an array of disks (Figure 2c). In the [Pt/CoFeB/MgO] multilayer, we defined a single square (Figure 2b), vertical stripes (Figure 2d), and an array of disks (Figure 2f).

We imaged both patterns in [Co/Pt] through a field sweep (positive to negative fields) and retrieved a hysteresis from the images of the combined reversal in ion‐irradiated and non‐irradiated regions (Figure 2a,c). We also display the magnetic hysteresis of the non‐modified [Co/Pt] (gray curve) for comparison. We observe an earlier onset of the domain nucleation in the irradiated regions of the film for both patterns. In panel c, the first slight reduction of the magnetization at an applied field of approximately 50 mT corresponds to the nucleation of a domain in the irradiated circles (Figure 2e). In addition, the domain width is significantly reduced in irradiated regions of [Co/Pt], as well as in the irradiated region of [Pt/CoFeB/MgO] shown in Figure 2b. We can only detect the irradiated areas by their altered magnetic properties resulting in domain configurations modified in alignment and domain size.

Moreover, local ion irradiation not only affects the size distribution of magnetic domains, but also guides and shapes the resulting magnetic domain alignment if the dimensions of the irradiated region are comparable or smaller than the domain width. In Figure 2d, the domains have aligned to the irradiation pattern of vertical stripes. In each of the visible three stripes, a vertical stripe domain is formed on the irradiated area's left and right edge. Going to round shapes, Figure 2c (inset) and f reveal a fully closed‐loop domain within each irradiated disk, remaining stable near zero magnetic field, surrounded by disordered stripe domains. Note that the ring domain is closed and surrounds a darker region of opposite magnetization, corresponding to a skyrmionium. At a magnetic field of µ_0_ *H*_z_ = 5 mT in Figure 2e, all irradiated disks in the field of view (FOV) are occupied by fully closed‐loop domains while spontaneous nucleation in the non‐irradiated magnetic film remains suppressed, offering a pathway for their selective synthesis in a homogeneously magnetized background.

### 3.2 Field‐Driven Formation

We investigated the field‐driven formation of these closed circular states through a combination of in situ STXM under variable magnetic fields and micromagnetic simulations of the magnetic textures. In both cases, we start from a uniformly magnetized state and gradually reduce the bias field while taking snapshots of subsequent magnetic configurations.

**Figure 3:**
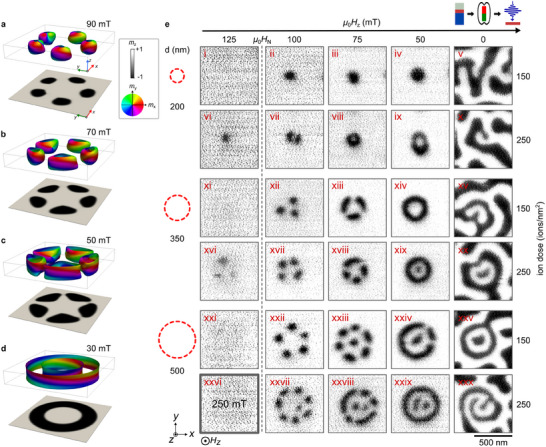
Field‐induced stabilization of higher‐order skyrmions in a [Pt/CoFeB/MgO] multilayer system. a–d) Micromagnetic simulations of the field‐driven nucleation process in an engineered disk with *d*_disk_ = 350 nm from (a–c) a flower state to a (d) 2π‐skyrmion. Isosurfaces correspond to in‐plane magnetization *m*_x, y_, and the out‐of‐plane magnetization *m*_z_ is shown underneath. e) STXM images of magnetic spin texture evolution under an out‐of‐plane magnetic field sweep from the saturation field µ_0_ *H*_z_ = 250 mT to zero field, for various disk diameters and ion doses. We created patterns in regions of 20μm × 20μm size each with different disk diameters *d*_disk_ ∈ {200, 350, 500 nm} with a spacing of 350 nm in between disks, and for ion doses of 150 ions/nm^2^ and 250 ions/nm^2^. Skyrmionium (xiv), target skyrmion (xix) and a skyrmion bag (xxix) coexist at µ_0_ *H*_z_ = 50 mT.

The simulated states in **Figure** **3**a–d correspond to the selected FOV of a single disk defect as illustrated in Figure 1h. The texture formation starts with the nucleation of individual bubble domains at the edge of the anisotropy‐engineered disk (Figure 3a). These bubbles contain Bloch defects in their domain walls. As the magnetic field is further reduced, the bubbles elongate along the edge of the disk shape (Figure 3b,c). Eventually they merge to form a closed‐loop domain (Figure 3d) with a defect‐free domain wall, which we identify as a 2π‐skyrmion. The full set of snapshots is available in Movie S1 (Supporting Information). In our simulations, we found that this formation process can be simulated without the presence of DMI and the magnetic texture is stabilized by stray fields only.

In our experiments presented in Figure 3e, we investigate this field‐driven formation in a [Pt/CoFeB/MgO] film which is magnetically structured with multiple arrays of helium‐ion‐irradiated disks (see Figure captions for details). In analogy to the simulation, the presented STXM images in Figure 3e are cropped to a specific region where we found textures that we consider typical for the field‐induced evolution. We started at a magnetic field of µ_0_ *H*_z_ = 250 mT, where the magnetic film is homogeneously magnetized (not shown). First textures appear when reducing the applied field to µ_0_ *H*_z_ = 125 mT for areas that have been irradiated with a larger ion dose of 250 ions/nm^2^. In the areas with smaller dose, the nucleation is detected at µ_0_ *H*_z_ = 100 mT. In agreement with the simulations, the textures nucleate as single skyrmions evenly distributed along the edge of the irradiated disk, with the number of skyrmions increasing with larger diameters of the disk. We introduce these configurations as flower states. The magnetic contrast of the textures nucleated at a higher magnetic field (panels vi and xvi) is much lower than those existing at lower fields, indicating a deviation from a columnar geometry, as predicted by the simulations in Figure 3a.

As the magnetic field is reduced further, the skyrmion bubbles elongate along the circular outline of the disk until the ring closes. For the larger diameters, the anisotropy‐engineered area is large enough for one or more additional skyrmions to form at the center. By choosing the diameter of irradiated disks, we can selectively induce the formation of either a skyrmionium (ix and xiv), a target skyrmion (xix), or a skyrmion bag (xxix). Remarkably, these higher‐order skyrmions coexist within the same ferromagnetic host material and stabilize under the same bias field conditions. Of all irradiated disks imaged in the field sweep, the success rate for creating a closed‐loop domain at µ_0_ *H*_z_ = 50 mT is 100% at the higher dose, whereas it is only 30% for the lower ion dose, determined from 54 nucleation attempts. We attribute minor deviations in the roundness of the skyrmion bags to variations in the underlying pinning landscape.

At zero magnetic field, magnetic domains also nucleate in the surroundings of the irradiated disks in a disordered manner. These domains partially merge with the texture in the disks. The presence of an applied bias field is, therefore, crucial for the stabilization of higher‐order skyrmions.

### 3.3 Controlled Laser‐Induced Nucleation

**Figure 4:**
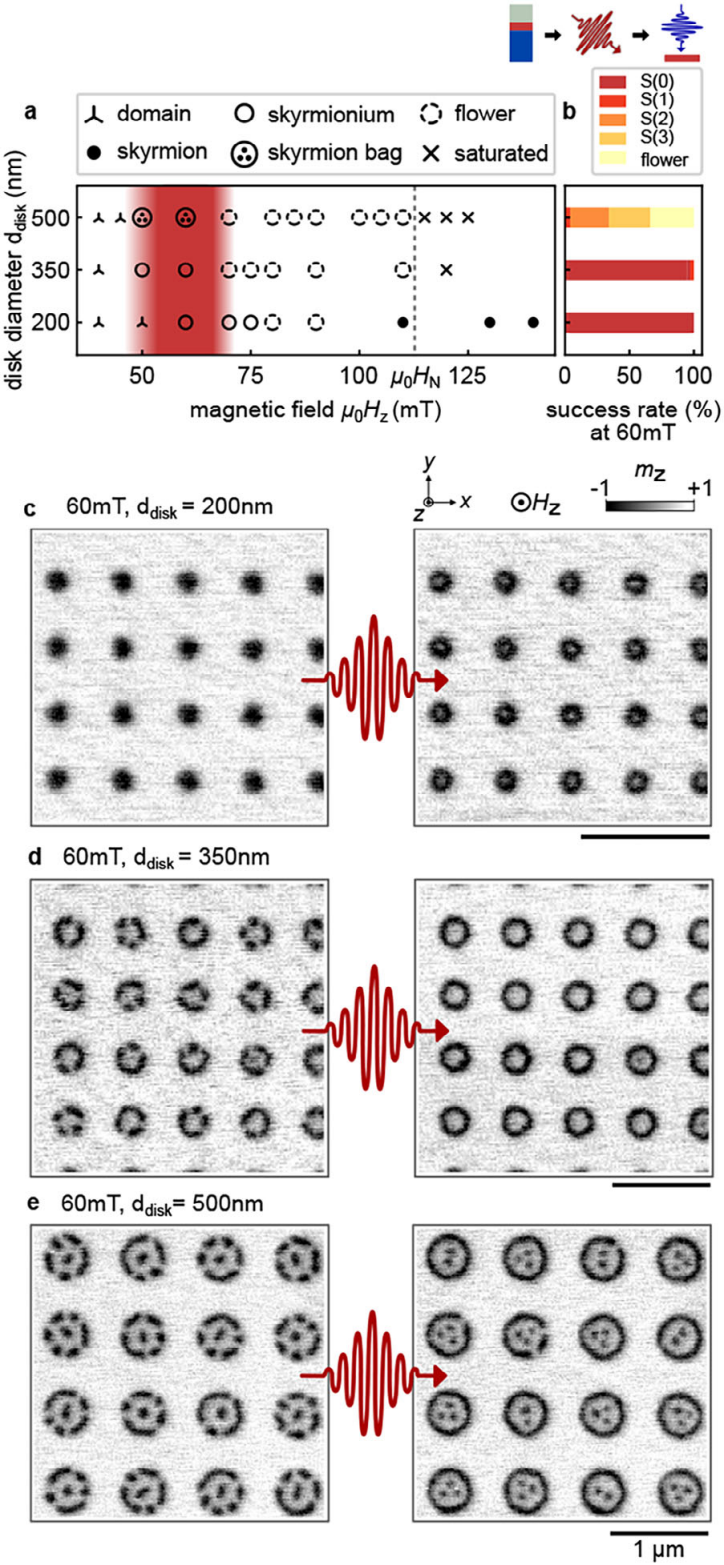
Laser‐induced nucleation of skyrmion bags. a) Phase diagram of induced spin textures after single IR‐pulse excitation as a function of µ_0_ *H*_z_ and *d*_disk_, as retrieved from STXM images. b) Success rate of laser‐induced nucleation at 60 mT. c–e) Representative STXM images depicting the nucleation of (c) skyrmionia, (d) skyrmionia and target skyrmions, and (e) skyrmion bags, displayed in the initial state before the IR‐pulse (left panel) and final state after the pulse (right panel). Scalebars correspond to 1μm.

To further control the formation of higher‐order skyrmions, we explore their generation using ultrafast infrared (IR) laser pulses in **Figure** **4**. After saturation and in presence of a bias field, we apply a single IR laser pulse with a 1039 nm wavelength, 1 ps pulse duration, and a focal spot size of 6.5μm (full width at half maximum, FWHM).

We found that two parameters are most critical for the synthesis of higher‐order skyrmions upon laser stimulus in our ferromagnetic multilayer system: the applied field *H*_z_ and the disk diameter *d*_disk_ of the ion‐irradiated regions. Notably, variations in laser fluence above the nucleation threshold of 5 mJcm^−2^ for our [Pt/CoFeB/MgO] multilayer^[^ ^23^, ^31^ ^]^ exhibited negligible impact on the formation of these higher‐order skyrmions. Also, we could not detect any difference between the two ion doses used in the experiment. The shapes of the nucleated textures are summarized in the phase diagram of the two critical parameters in Figure 4a, where each data point represents the average of up to eight repetitions of the same experimental procedure. Specifically, we first saturate the sample at µ_0_ *H*_z_ = 250 mT, reduce the field, capture the initial magnetic state, apply a single IR laser pulse, and then record the resulting magnetic configuration. The data points reflect these final configurations imaged with STXM (see legend in the Figure 4a).

When reducing the magnetic field from positive saturation to around µ_0_ *H*_z_ = 150 mT, both the non‐irradiated and irradiated regions of the magnetic film remain homogeneously magnetized, suppressing laser‐induced nucleation entirely. However, for the smallest disk diameter of 200 nm, laser‐induced skyrmion nucleation in the irradiated disk becomes possible in a reduced field range from µ_0_ *H*_z_ = 140 mT to 110 mT. This finding aligns well with previous observations.^[^ ^27^ ^]^ Further reduction in the applied field below the nucleation field µ_0_ *H*_N_ leads to spontaneous texture formation in the irradiated disk as already shown in Figure 3e. Considering patterns irradiated with an ion dose of 150 ions/nm^2^, predominantly flower states with varying number of skyrmions (1–3) are observed depending on the size of the ion‐irradiated circle and the applied field, similar to the states presented in Figure 3. In a wide field range from µ_0_ *H*_N_ to ≈70 mT, these textures remain mostly unaffected by single‐pulse laser excitation. We only occasionally find small rearrangements of the flower configuration with skyrmions added to the texture (not shown). However, in a distinct range of applied field from ≈70 mT to ≈50 mT (marked in red), the laser excitation can transform single skyrmions and flower states into bags of different order (S(0)–S(4)) depending on the size of the disk. The formation of 2π‐skyrmions out of π‐skyrmions in disks with *d*_disk_ = 200 and 350 nm is illustrated in Figure 4c,d. For the latter size, we also observe the formation of a 3π‐skyrmions. However, 3π‐skyrmions can also occassionally form in 500 nm disks (not shown). Skyrmion bags with two to three central skyrmions typically emerge in *d*_disk_ = 500 nm disks (Figure 4e).

In the optimal applied field range around µ_0_ *H*_z_ = 60 mT as highlighted in the phase diagram in Figure 4a, all different types of higher‐order skyrmions can be created via laser excitation with very high fidelity. We observe a success rate of finding a closed circular domain of 70–100 %, depending on the disk diameter (Figure 4b), determined from 304 nucleation attempts. The textures appear isolated without any nucleation of domains in the non‐ion‐irradiated regions of the film. However, reducing the field below *H*_z_ = 50 mT triggers the spontaneous nucleation of magnetic domains, which progressively disrupts and fragments the higher‐order skyrmionic textures.

### 3.4 Size‐Dependent Stabilization of Higher‐Order Skyrmions

**Figure 5:**
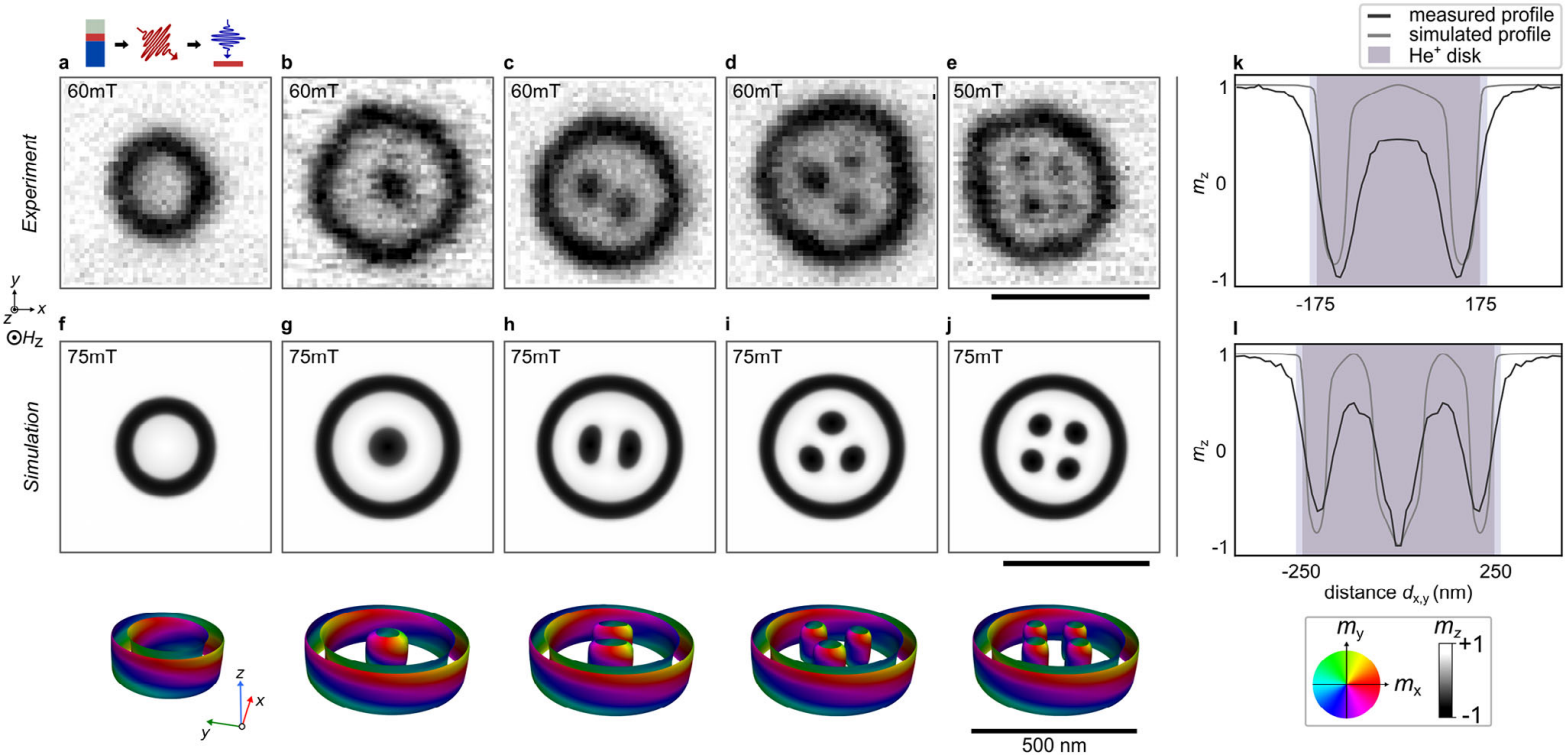
Variety of stable skyrmion bags in experiment and simulation. a–e) top: Representative STXM images proving the laser‐induced formation of (a) skyrmionium (or *S*(0)‐bag), (b) target skyrmion (or *S*(1)‐bag), (c) *S*(2)‐bag, (d) *S*(3)‐bag and (e) *S*(4)‐bag. f–j) Numerical simulations of corresponding skyrmion bags. center: Averaged OOP‐magnetization, *bottom*: Isosurfaces of the skyrmion states, defined by *m*_*z*_ = 0. The disk diameter was (a,f) *d*_disk_ = 350 nm and (b‐e, g‐j) *d*_disk_ = 500 nm. The applied ion dose in (a‐e) was 150 ions/nm^2^. The laser fluence was 9 mJcm^−2^. k–l) Magnetization profiles for (k) skyrmionium from (a) and (f), and (l) target skyrmion from (b) and (g).

Our systematic investigation reveals a strong correlation between the disk diameter and the type of stable skyrmionic textures that can be supported. In the smallest irradiated areas (200 nm diameter), we exclusively observe 2π‐skyrmion states. Similarly, in 350 nm diameter areas, 2π‐skyrmion states dominate, appearing in approximately 95 % of all cases following laser excitation (**Figure** **5**a,b). This preferential formation of 2π‐skyrmion states in smaller disks suggests a critical size threshold for stabilizing more complex magnetic configurations.

The transition to higher‐order skyrmion bags (*S*(*Q*), *Q* > 0) requires larger disk diameters. In 500 nm disks, we observe a diverse range of stable configurations, including target skyrmions (*S*(1)) and higher‐order skyrmion bags up to *S*(4) (Figures 5b–e). Notably, skyrmionium states (*S*(0)) are absent in these larger disks, indicating a size‐dependent selection mechanism for specific magnetic textures. Micromagnetic simulations (detailed in Methods) corroborate these experimental findings (Figures 5f–j). In 500 nm disks, we successfully stabilize all four experimentally observed states. However, in modeling, stable skyrmionium configurations only emerge when the disk diameter is reduced to 350 nm. The 3D magnetization maps from our simulations reveal continuous spin winding within the closed‐loop domains, characterized by defect‐free domain walls.

A particularly interesting feature emerges in higher‐order skyrmion bags: as the number of embedded skyrmions increases, their individual sizes decrease. This size reduction additionally manifests experimentally as diminished contrast in smaller skyrmions, primarily due to the limited spatial resolution of the STXM measurements. The experimental data also captures the regular geometric arrangement of inner skyrmions, reflecting the strong stray‐field interactions characteristic of our ferromagnetic material system.

To better understand the spatial extent of these magnetic textures, we analyzed the magnetization profiles of centrosymmetric skyrmionium and target skyrmion states in relation to the He^+^‐irradiated disk boundaries (Figures 5k,l). In our simulations, the disk boundary is represented by a sharp step in magnetic anisotropy. While the outer domain wall of the closed‐loop domain closely follows this anisotropy step, it remains predominantly confined within the region of reduced anisotropy. Experimentally, we observe that the domain wall extends slightly beyond the nominal irradiated area. This discrepancy can be attributed to two factors: the limited spatial resolution of our imaging technique and the lateral spread of the ion cascade inside the material, which likely extends the effectively modified region by approximately 10 nm beyond the nominal irradiation boundary^[^ ^27^ ^]^ (indicated by the lighter shading of irradiated area in the plots).

We also observe a reduction in magnetic contrast for both skyrmionium and target skyrmion textures compared to the saturated film state. This contrast reduction can be explained by three mechanisms: i) a slight decrease in saturation magnetization due to ion irradiation, although this effect is limited to approximately 4 % as measured in fully field‐polarized films; ii) the finite spatial resolution of STXM imaging; and iii) an in‐plane tilt of magnetic moments, particularly pronounced at the top and bottom boundaries, resulting in broader domain walls. The combination of these effects contributes to the observed contrast variations in our experimental measurements.

## 4 Discussion

The stabilization of higher‐order skyrmions—including skyrmionium, target skyrmions, and skyrmion bags—in laterally unconfined ferromagnetic thin films represents a pivotal advancement in controlling and understanding complex magnetic textures. The observation of these complex spin textures at room temperature, without requiring topographic confinement, represents a significant advance for potential applications. The skyrmion bags emerge as the local ground state in ion‐irradiated disks, exhibiting stability comparable to skyrmions or domains in these films at room temperature. Our quasi‐static measurements require stability on timescales of at least seconds to minutes. We have never observed any modifications or annihilation of bags during image acquisition that would manifest themselves as sudden (non‐physical) changes in the magnetization within the STXM scanning pattern, such as line defects.

A remarkable finding is the successful generation of higher‐order skyrmions in both symmetric [Co/Pt] and asymmetric [Pt/CoFeB/MgO] multilayers. The consistent formation of closed‐loop domains in both systems suggests that the Dzyaloshinskii–Moriya interaction (DMI) plays a minor role in stabilizing these textures, as DMI is expected to be present only in the asymmetric material stack. Instead, our experimental and simulation results indicate that dipolar interactions primarily govern their stability.

However, DMI is known to energetically favor a specific chirality, leading to the formation of homochiral topological configurations, such as skyrmions.^[^ ^23^ ^]^ Our [Pt/CoFeB/MgO] host material is therefore particularly well‐suited for technological implementation, featuring low pinning,^[^ ^70^ ^]^ and compatibility with spin‐orbit torque (SOT)‐driven motion.^[^ ^13^, ^19^, ^27^ ^]^ Moreover, our anisotropy‐engineering approach can be readily adapted to various material platforms, offering broad applicability.

Our investigation reveals two distinct mechanisms for higher‐order skyrmion formation: The first mechanism relies on domain nucleation and transformations out of the field‐polarized state when lowering the applied field. Skyrmion bubble states emerge at the disk edges, forming intermediate “flower states” that may contain an inner skyrmion depending on the disk diameter. Further field reduction transforms these flower states into closed‐loop domains through Bloch point‐mediated merging of domain ends. The anisotropy step appears to act as a barrier to radial domain wall motion, while stray fields enforce equidistant spacing between bubbles in the flower states. At lower fields, the energetic landscape favors domains that merge within the disk rather than expand beyond it. Micromagnetic simulations confirm this mechanism, demonstrating how the combination of local anisotropy modifications and external fields creates an energy landscape that stabilizes higher‐order skyrmion bags.

The laser‐induced mechanism offers an alternative route: a single laser pulse can transform a field‐stabilized flower state into a higher‐order skyrmion. The final configuration—either an empty or filled skyrmion bag—depends on the diameter of the irradiated area. At moderate applied fields (60 mT), the ion‐irradiated regions exhibit locally reduced thresholds that enable topological transformations under sufficient laser fluence while the unmodified film remains below the optical nucleation threshold.^[^ ^23^ ^]^ This process demonstrates remarkable reliability and high success rates independent of the ion dose in the range from 150 to 250 ions/nm^2^. Notably, the number of skyrmions nucleated inside the bag varies stochastically—ranging from zero to three for disks with 500 nm diameter (Figure 4b), with an exceptional S(4) bag observed once (Figure 5e). Despite homogeneous ion irradiation across all disks, this variation persists between trials on identical structures. The underlying mechanism involves laser‐induced ultrafast heating that creates a high‐temperature fluctuation state where skyrmions continuously nucleate and annihilate, as demonstrated in earlier work.^[^ ^23^ ^]^ During the subsequent cool‐down phase, a random number of skyrmions “freeze out” at stochastic positions within the irradiated region, which acts as a preferential nucleation site compared to non‐irradiated areas. The ability to control higher‐order skyrmion nucleation using ultrafast laser pulses presents exciting opportunities for technological applications requiring rapid and precise magnetic switching.

## 5 Conclusion

We have demonstrated the controlled creation of higher‐order skyrmions in room‐temperature ferromagnetic thin films, achieving the synthesis of isolated *S*(0), *S*(1), *S*(2), *S*(3) − *S*(4)‐skyrmion bags in laterally unconfined geometries and overcoming the longstanding challenge of experimentally stabilizing these higher‐order skyrmion configurations. Through precise anisotropy engineering and disk diameter control, we established a protocol for reliable site‐specific nucleation and selection of specific skyrmion states. The circular geometry of the irradiated disks proves particularly effective in facilitating closed‐loop domain formation, highlighting the crucial role of the anisotropy step and the importance of defect design to stabilize higher‐order topological states. The demonstrated highly efficient laser‐induced nucleation process, achieving near‐perfect conversion rates, establishes a robust platform for ultrafast generation of these complex magnetic textures.

Looking ahead, our findings open promising research directions. We provide a reliable platform for investigating the topology‐dependent lifetime and robustness of the bags with respect to temperature, changes in the applied field, or other stimuli like current‐induced spin‐orbit torques. The potential to mobilize higher‐order skyrmions and study their interactions^[^ ^39^, ^57^, ^61^, ^65^ ^]^ could reveal novel functionalities for spintronic applications.^[^ ^34^, ^65^ ^]^ Their compatibility with ultrafast optical manipulation and the ability to encode multiple states within a single material system makes them particularly attractive for high‐density storage and high‐speed switching applications.^[^ ^67^ ^]^ These capabilities position higher‐order skyrmions as versatile building blocks for next‐generation magnetic information technologies, bridging fundamental research and practical applications.

## 6 Experimental Section

### Nanofabrication

Initially, magnetic multilayers were prepared with a nominal structure of Ta(3 nm)/ [Co(0.6 nm)/ Pt(0.8 nm)]12/ Ta(2 nm) and Ta(3 nm)/ Pt(4 nm)/ [Pt(2.5 nm)/Co_60_Fe_24_B_16_(0.72 nm)/ MgO(1.4 nm)]_15_/ Pt(2 nm), deposited on 150 nm‐thick silicon nitride membranes using argon‐assisted DC and RF magnetron sputtering. Here, these multilayers were referred to as [Co/Pt] and [Pt/CoFeB/MgO], respectively. Both material systems had perpendicular magnetic anisotropy and were known to support the generation of π‐skyrmions via field cycling^[^ ^71^ ^]^ and laser excitation.^[^ ^23^, ^31^ ^]^

Second, focused He^+^‐ion irradiation was locally applied to the magnetic material in various patterns and doses, utilizing a ZEISS Orion NanoFab He^+^‐FIB system with the FIB‐o‐mat software package.^[^ ^72^ ^]^ The estimated focal spot size of the He^+^‐FIB was around 1 nm, with a pattern placement accuracy below 10 nm, setting the nominal resolution for FIB patterning. However, the actual extent of material modification was influenced by the spatial spread of the collision cascade within the material and the accompanying damage, which broadens the effective beam radius along the depth of the magnetic multilayer, estimated by approximately 10 nm.^[^ ^27^ ^]^ In this manuscript, ion pattern dimensions were reported as the intended target sizes of the irradiated areas. The irradiated areas were constructed from filled rectangular and circular shapes, rasterized with a dwell time of 0.1μs and a pitch of 1 nm along the serpentine (rectangular shape) or spiral (circular shape) beam path. In all presented arrays of circular areas, the circles were spaced by 350 nm in *x* and *y* direction. The patterning was carried out at an acceleration voltage of 30 kV, using an aperture width of 20μm, a gas flow of 2 × 10^−6^ Torr, and spot control settings between five and six,^[^ ^72^ ^]^ resulting in ion currents of 2.5 to 4 pA. Upon ion irradiation, intermixing at the interfaces and atomic displacements throughout the material stack lead to an overall reduction in PMA.^[^ ^73^, ^74^ ^]^ In this films, this reduction of PMA manifests itself by a reduction of the characteristic domain width as expected from the competing interactions in such multilayers, see, e.g., Ref. [75]. Magnetic imaging confirmed that there were no permanent changes in magnetic contrast at irradiated locations in magnetically saturated samples.

The measurements presented in Figures 3 and 4 were obtained from the same sample to ensure comparability for field‐ and laser‐induced nucleation. On this sample, six grids had been prepared, each measuring 20μm × 20μm. Each grid corresponds to a specific combination of disk diameter and ion dose, with disks separated by 350 nm. Depending on the diameter, each grid contains between 324 disks (largest) and 900 disks (smallest). For the field‐driven formation, hysteresis data sets were recorded for six combinations of disk diameter and ion dose, as presented in Figure 3, with nine dots per image, yielding 54 nucleation attempts in total. For the controlled laser‐induced nucleation, 75 images were recorded after laser excitation on field of views with a varying number of nine to 16 dots, yielding 304 nucleation attempts in total. In the imaging experiments, field of views were selected at random positions on the grids and highlight representative, high‐resolution images in the Figures.

### Nanoscale Imaging

*Magnetic Force Microscopy*

Magnetic force microscopy was performed at Max Born Institute, Berlin, using a Bruker Dimension Icon instrument. Prior to the measurement, samples were demagnetized in an out‐of‐plane alternating magnetic field, decaying from 1.2T to zero field. Images of the topography of the samples and its magnetization at zero magnetic field were acquired with a resolution of below 30 nm, defined by the dimensions of the tip, as presented in Figure 2b, df. The tips used were Nanosensors PPP‐LM‐MFMR (Tip radius < 30 nm, coercivity 25 mT, remanent magnetization 150 emu cm^−2^). The MFM images in Figure 2b,d,f had been postprocessed applying data correction features within the Gwyddion framework, including a Gaussian Filter (σ = 3 px) to reduce artefacts. Note, however, the softness of the material facilitates interactions with the tip, potentially leading to minor jumps in the image. The topographic profile did not show any permanent change due to the local ion irradiation, confirming previous findings.^[^ ^27^ ^]^

*X‐ray Imaging*

Magnetic textures were imaged using scanning transmission x‐ray microscopy (STXM) at the MAXYMUS endstation^[^ ^76^ ^]^ of the BESSY II electron storage ring, operated by Helmholtz‐Zentrum Berlin für Materialien und Energie. This technique provided spatial resolution down to approximately 20nm. During imaging, a variable out‐of‐plane magnetic field (*H*_z_) was applied via a system of four rotating permanent magnets. Images were captured in transmission geometry using circularly polarized x‐rays tuned to the Co L_3_ absorption resonance at a photon energy of nominally 778.5 eV, allowing for magnetic contrast through the x‐ray magnetic circular dichroism (XMCD) effect. The resulting magnetization maps represent the sample's out‐of‐plane magnetization component, and all STXM images were shown as recorded within a single scan using a specific x‐ray helicity.

At the MAXYMUS microscope, an MBI‐developed, custom‐built picosecond fiber‐laser system (wavelength: 1039 nm, spot size: 6.5μm FWHM, pulse duration: 1 ps) enables in situ optical excitation of the sample.^[^ ^27^ ^]^

### Micromagnetic Modelling

Micromagnetic simulations based on an effective medium model (Figure 1f) were conducted to investigate the magnetic behavior of the multilayer structures. This approach enables the analysis of multilayers with varying layer thicknesses and materials, significantly reducing the computational complexity typically required to model each layer individually. The simulations were performed using MuMax3,^[^ ^77^ ^]^ which solved the Landau‐Lifshitz‐Gilbert equation to model the evolution of magnetization over time. The parameters used in the simulations were effective medium parameters, given by *M*_s_ = 177 kAm^−1^, *A* = 0.19 pJm^−1^, and *K* = 30 kJm^−3^. For these simulations, a system size of 1000 × 1000 × 78.2 nm^3^ was considered, discretized into 512 × 512 × 32 cells, chosen to contain a single irradiated disk. Within the disk, the anisotropy was set to 20% of the CoFeB anisotropy in line with prior studies. Significance for DMI presence was not observed, which was reported previously,^[^ ^10^ ^]^ in reproducing the nucleation and stability of skyrmion bags observed in the experiment. That sufficiently expanded the range of magnetic systems, including centrosymmetric bubble materials that could host various skyrmion bags.

To reproduce skyrmionium nucleation within a 350 nm disk, the system was incrementally relaxed from the saturated state, starting at *B* = 200 mT and reducing the field in steps of Δ*B* = 5 mT. Consistent with experimental results, initial nucleation of skyrmions (or bubbles with *Q* = −1) occurred along the disk edge. As the field was further reduced, the skyrmions merged, eventually forming a skyrmionium state (*Q* = 0). During this topological transition, the simulations revealed the emergence of Bloch points, which could escape the system through top and bottom boundaries. Notably, the mobility of the Bloch points strongly depends on the value of the exchange stiffness along the film thickness. In particular, at *A*_*z*_ = 0.5*A*, a stable skyrmionium state free of Bloch points was obtained.

To test the stability of skyrmion bags (Figure 5) in a system with a 500 nm irradiated disk, the system was relaxed at *B* = 75 mT starting from appropriate ansatz states.^[^ ^41^ ^]^ Directly simulating the nucleation process of these states was challenging, as the simulations did not account for effects such as finite temperature, surface nonuniformity, or anisotropy variations, which were inevitably present in experimental conditions. Evidence of the presence of these effects can be seen in Figures 3 and 5, where the number of nucleated skyrmions varies randomly among disks of identical size.

## Conflict of Interest

The authors declare no conflict of interest.

## Author Contributions

L.‐M.K. and B.P. conceived the experiments. L.‐M.K., V.D., M.S., C.M.G., D.E., and K.H. prepared the samples. L.‐M.K., C.K., T.S., M.A., S.G., K.G., R.B., St.W., T.K., I.W., S.W., and M.W. performed the experiments. V.M.K. performed the micromagnetic simulations. L.‐M.K. analyzed the data and prepared all figures. L.‐M.K., V.M.K., F.B., S.E., and B.P interpreted the results. All authors reviewed and commented on the manuscript.

## Supporting information

Supplemental Movie S1

Controlled Formation of Skyrmion Bags

## Data Availability

The data that support the findings of this study are available from the corresponding author upon reasonable request.
