# Supplementary material for: Controlled Formation of Skyrmion Bags
Source: Adv Mater. 2025 Apr 21;37(29):2501250. doi: 10.1002/adma.202501250 (PMC12288806; doi:10.1002/adma.202501250)
Supplement: Supplementary file 2 — Controlled Formation of Skyrmion Bags [file ADMA-37-2501250-s002.pdf]

ADVANCED MATERIALS

Supporting Information

for *Adv. Mater.*, DOI 10.1002/adma.202501250

Controlled Formation of Skyrmion Bags

Lisa-Marie Kern, Vladyslav M. Kuchkin, Victor Deinhart, Christopher Klose, Themistoklis Sidiropoulos, Maike Auer, Simon Gaebel, Kathinka Gerlinger, Riccardo Battistelli, Steffen Wittrock, Tamer Karaman, Michael Schneider, Christian M. Günther, Dieter Engel, Ingo Will, Sebastian Wintz, Markus Weigand, Felix Büttner, Katja Höflich, Stefan Eisebitt and Bastian Pfau*

Supporting Information to: Controlled Formation of Skyrmion Bags

Lisa-Marie Kern,[*,†] Vladyslav M. Kuchkin,[‡] Victor Deinhart,[¶,§] Christopher Klose,[†] Themistoklis Sidiropoulos,[†] Maike Auer,[†] Simon Gaebel,[†] Kathinka Gerlinger,[†] Riccardo Battistelli,[§,‖] Steffen Wittrock,[§] Tamer Karaman,[‖] Michael Schneider,[†] Christian M. Günther,[⊥] Dieter Engel,[†] Ingo Will,[†] Sebastian Wintz,[§] Markus Weigand,[§] Felix Büttner,[§,‖] Katja Höflich,[¶] Stefan Eisebitt,[†,#] and Bastian Pfau[†]

†*Max Born Institute for Nonlinear Optics and Short Pulse Spectroscopy, Berlin, 12489, Germany*

‡*Department of Physics and Materials Science, University of Luxembourg, L-1511 Luxembourg, Luxembourg*

¶*Ferdinand-Braun-Institut (FBH), Berlin, 12489, Germany*

§*Helmholtz-Zentrum Berlin für Materialien und Energie GmbH, Berlin, 14109, Germany*

‖*Experimental Physics V, Center for Electronic Correlations and Magnetism, University of Augsburg, 86159 Augsburg, Germany*

⊥*Technische Universität Berlin, Zentraleinrichtung Elektronenmikroskopie, Berlin, 10623, Germany*

#*Technische Universität Berlin, Institut für Optik und Atomare Physik, Berlin, 10623, Germany*

E-mail: kern@mbi-berlin.de

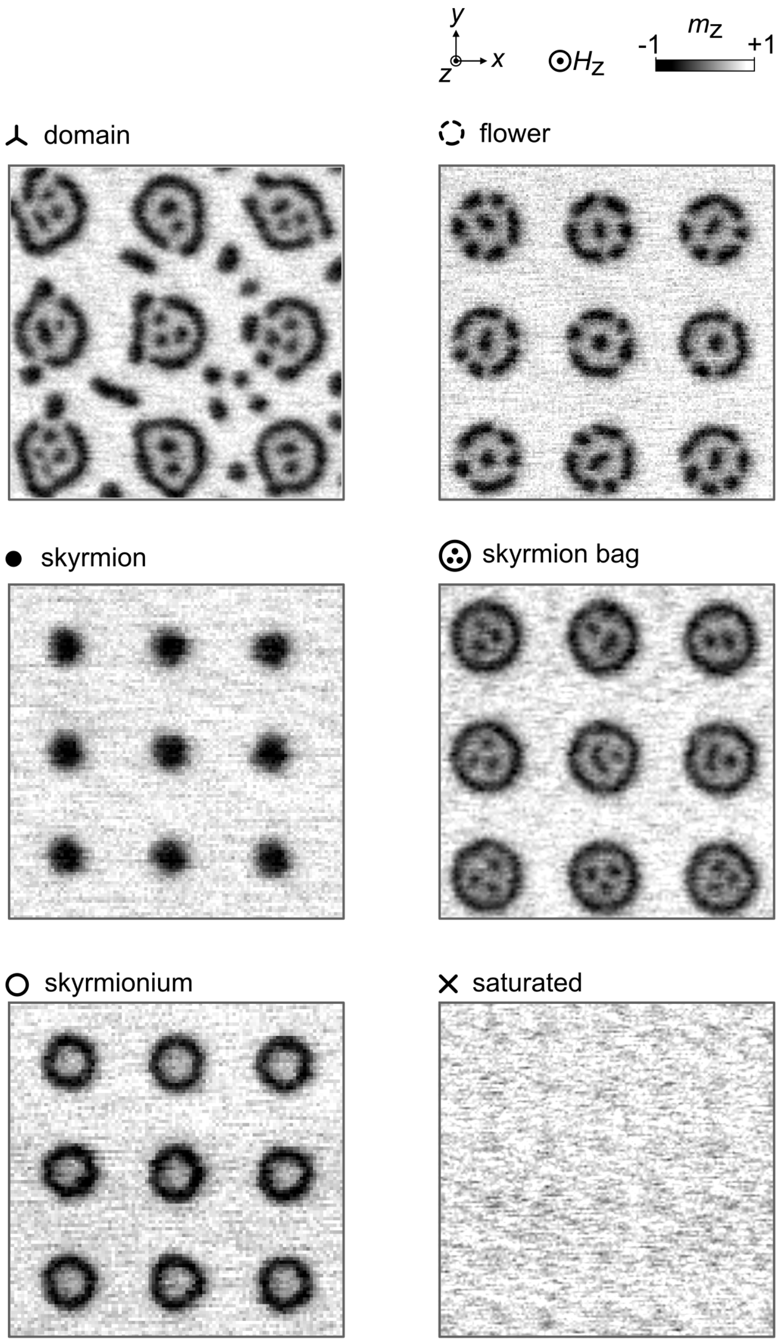

Figure S1: The STXM images represent the final configurations in the phase diagram and illustrate the legend symbols used in Fig. 4 of the main text.
